# The Structural Proteins of Thermophilic Bacteriophage P23-77: Expression and Characterization

**DOI:** 10.3390/ijms26178688

**Published:** 2025-09-06

**Authors:** Milad Kheirvari, Ebenezer Tumban

**Affiliations:** Graduate Program in One Health Sciences, School of Veterinary Medicine, Texas Tech University, Amarillo, TX 79106, USA; milad.kheirvari@ttu.edu

**Keywords:** thermophilic bacteriophage P23-77, coat proteins, membrane-associated proteins, protein expression and purification, 3D structure prediction, *Thermus thermophilus*, BL21 star

## Abstract

P23-77 is a thermophilic bacteriophage that infects *Thermus thermophilus* bacteria. The genome of the virus is enclosed in an icosahedral capsid. This capsid is made of the small major capsid protein (VP16), the large major capsid protein (VP17), and the minor capsid protein (VP11). In addition to these three structural proteins, membrane-associated proteins (VP15, VP19, VP20, VP22, and VP23) have been identified in the virus and may serve as scaffold proteins to help with viral assembly. Previous studies have expressed VP11, VP16, and VP17 in *E. coli*. A mixture of these proteins can lead to the formation of complexes. However, the potential to express membrane-associated proteins has never been explored. Here, we demonstrated, for the first time, the expression and co-expression of some membrane-associated proteins with capsid (coat) proteins, both in the natural host and in *E. coli*. Co-expression of these proteins did not result in the assembly of virus-like particles. We explored further strategies to express and purify some of the proteins for future studies. We observed that the insertion of a purification tag (Strep-II tag, but not a histidine tag) significantly reduced the expression levels of some of the proteins. Six of the eight structural proteins were successfully purified to homogeneity using different approaches. We showed that VP20 and VP22 migrated on SDS PAGE gel at sizes larger than their predicted molecular weights. Predicted 3D structures of the proteins show that most of them are helical in nature with disordered regions. The work presented here will help pave the way for the expression and purification of these proteins. This will help determine their 3D structures and may shed light on the requirements for viral assembly.

## 1. Introduction

P23-77 is a thermophilic bacteriophage that was isolated (Promega collection) from an alkaline hot spring in New Zealand [1]. The virus infects *Thermus thermophilus* bacteria, which have an optimal growth temperature of 70–75 °C [1,2,3]. The genome of the virus, double-stranded circular DNA (~17 Kbp), encodes both structural and non-structural proteins [2,3]. While the functions of most of the non-structural genes in the genome (topoisomerase, ATPase, lysozyme, endolysin, transglycosylase, etc.) have been identified, the functions of most of the structural genes are not known. The genome is enclosed in an icosahedral capsid, which is composed of three proteins: two major coat proteins [the small major capsid protein (VP16) and the large major capsid protein (VP17)] and a minor capsid protein VP11 [3,4,5]. VP11 is a ~22.06 KDa protein, VP16 is ~19 Kda, while VP17 is a ~32 Kda protein. It is estimated that the viral capsid is assembled from 147 copies of VP11, 1080 copies of VP16, and 540 copies of VP17 [4,5]. The capsid of this bacteriophage is 6 nm thick and has a high GC content (including the genome) of greater than 66% [2,6]. These features make P23-77 highly thermostable; in fact, studies have shown that all three coat proteins can withstand temperatures above 80 °C [4,5,7]. These features make proteins derived from P23-77 attractive for biomedical applications. For example, thermophilic enzymes derived from other thermophilic bacteriophages have been used in the polymerase chain reaction to amplify genes and in the agricultural industry to control the growth of mesophilic bacteria (reviewed in [6]). On the other hand, structural proteins from thermophilic bacteria (unlike those from mesophilic bacteria) have not been explored for biomedical applications. For example, structural proteins from bacteriophages can be used to develop virus-like particle (VLP) platforms for vaccine design.

VLPs are empty viral shells that may be formed naturally during the life cycle of a virus or can be obtained by cloning and expressing viral structural proteins, such as capsid (coat) protein(s) or envelope protein, in a host cell, including eukaryotic or bacterial cells. Following expression in the host cell, structural proteins, for example, coat protein(s), spontaneously self-assemble to form VLPs [8,9,10]. Thus, VLPs consist of one or more types of multimeric coat proteins arranged geometrically into dense, repetitive (multivalent) arrays. VLPs are morphologically and structurally similar to viruses from which the coat proteins are derived, except for the fact that they lack the viral genome. VLPs, if successfully developed from thermophilic structural proteins (e.g., from bacteriophage P23-77), would have several advantages compared to those developed from viruses that infect mesophilic bacteria. Firstly, for example, they are expected to be thermostable based on the features (high GC content and the thickness of the capsid) already mentioned above. Secondly, a high copy number of heterologous proteins can be displayed on VLPs from this virus. As mentioned above, the P23-77 viral capsid is believed to be assembled from 147 copies of VP11, 1080 copies of VP16, and 540 copies of VP17. An insertion of a heterologous antigen in any of the three coat proteins implies that the corresponding copies of the heterologous antigen will be displayed on the VLPs. Thirdly, bacteriophage P23-77 is 78 nm in diameter [2]. VLPs derived from this thermophilic bacteriophage are larger compared to other VLPs; a larger diameter implies that VLPs can be loaded with more vaccine adjuvants, imaging fluorophores, or cargo for targeted delivery to cells. The expression and purification of structural proteins from P23-77 can pave the way for determining the 3D structures of these proteins and consequently provide information on their function and how they interact with each other to form the viral capsid. As mentioned above, the capsid of this bacteriophage is made up of three proteins: VP11, VP16, and VP17. All three coat proteins have been cloned and expressed separately in *E. coli* bacteria; a mixture of all three purified coat proteins showed that they can form oligomeric complexes (aggregation/particle formation) in vitro [7]. In another study, Yadav [7] co-expressed these proteins in *E. coli*. Co-expressed proteins formed oval structures (with concentric cycles) but were smaller in size compared to the size of the virus. In addition to VP11, VP16, and VP17, other structural proteins have been identified in this virus to be associated with these coat proteins [3]. These are membrane-associated proteins and include VP15, VP19, VP20, VP22, and VP23. Membrane-associated proteins, in some viruses, serve as scaffold proteins that help with viral assembly (reviewed in [11]). The ability of these proteins to be expressed from an expression vector, their sizes confirmed experimentally, and purified for downstream assays has never been explored. Secondly, it has not been explored whether the co-expression of these membrane-associated proteins with VP11, VP16, and VP17 promotes the assembly of these proteins into VLPs. In this study, we evaluated the expression and co-expression of the coat proteins and membrane-associated proteins of P23-77 in both its native thermophilic host (*T. thermophilus*) and in strains of *E. coli*, with the goal of assessing their potential to self-assemble into VLPs. The use of a thermophilic expression system was intended to mimic the native conditions (metabolic activities/environment) of the viral host, such as temperature, ionic strength, and pH, that the virus normally replicates, and some of which promote the stability of the capsid (reviewed in [6]). In this study, we used HB27:nar thermophilic bacteria, which are a derivative of *T. thermophilus* HB27. HB27:nar bacteria contain the nitrate reductase (nar) operon, which allows it to grow under both aerobic and anaerobic conditions—providing flexibility in protein expression studies. On the other side, we also chose to use a mesophilic expression system, *E. coli*, for expression to simplify the production process and increase accessibility. *E. coli* is cost-effective, easy to engineer (e.g., promoter or antibiotic changes), and does not require additional chemicals for growth nor specialized high-temperature equipment as required by thermophilic bacteria; most laboratories do not have incubators capable of sustaining the high temperatures required for thermophilic hosts. Thus, expressing these proteins in *E. coli* allows for broader applicability and easier scalability of protein expression. In addition to co-expression, we optimized protein expression and purification of some of the proteins from *E. coli* lysates. We show that VP20 and VP22 migrated on SDS PAGE gel at sizes higher than their predicted molecular weights (based on amino acid sequences). The predicted 3D structures of the proteins show that most of them are helical in nature with disordered regions.

## 2. Results

### 2.1. VP11/VP16/VP17 Coat Proteins and VP15/VP19/VP20/VP22 Membrane-Associated Proteins Were Successfully Expressed in T. thermophilus HB27:nar but at Different Levels

**Figure 1:**
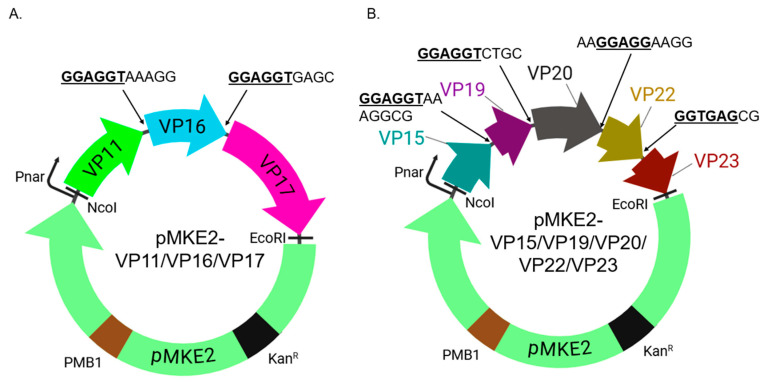
Design of the expression vectors of viral proteins (VPs) in HB27:nar bacteria. (**A**) Fragments VP11, VP16, and VP17 were cloned into pMKE2 plasmid using NcoI and EcoRI sites. (**B**) Fragments VP15, VP19, VP20, VP22, and VP23 were cloned into pMKE2 plasmid using the same restriction sites. Linker sequences between VPs as they appear in the genome are shown in capital letters. Native Shine–Dalgarno sequence (GGAGGT) in P23-77 genome between VPs is bolded and underlined. Pnar: nitrate reductase promoter. PMB1: origin of replication. Kan^R^: kanamycin antibiotic resistance gene.

The three coat proteins (VP11, VP16, and VP17) were cloned into the pMKE2 expression vector using NcoI and EcoRI sites (Figure 1A) while the five membrane-associated proteins (VP15, VP19, VP20, VP22, and VP23) were separately cloned into the same vector using the same restriction sites (Figure 1B). Linker sequences (with the native Shine-Dalgarno sequence) as they appear in the genome of the virus were included between the genes. In addition to this, a silent mutation (GCC to GCG) was introduced at amino acid 266 in VP17 to abolish an internal NcoI site in the gene; another silent mutation (ATA to ATC) was introduced at amino acid 154 in VP20 to abolish an internal NdeI site in the gene. To assess if the coat proteins and membrane-associated proteins could be expressed in the native host bacterium, we transformed the two plasmids separately into *T. thermophilus*.

**Figure 2:**
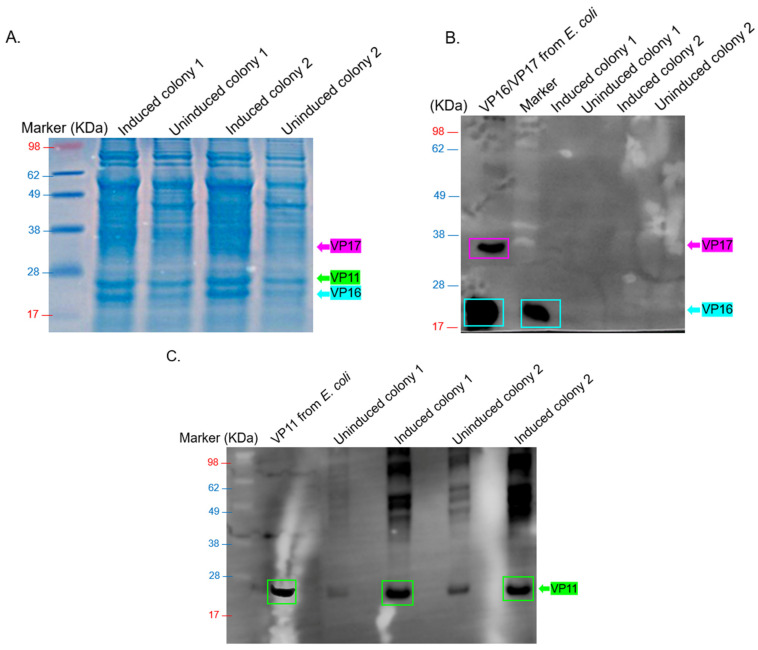
Expression of polycistronic construct (pMKE2-VP11/VP16/VP17) in *T. thermophilus* HB27:nar. (**A**) Bacterial lysates (from co-expressed VP11/VP16/VP17 culture induced with 40 mM KNO3 for 4 h) were separated on SDS PAGE gel and stained with Coomassie Blue. (**B**) Western blot of lysates from panel (**A**) was run on SDS PAGE. Protein was transferred to the membrane and probed with a mixture of anti-VP16 and anti-VP17 polyclonal sera (at 1:1000 dilution). (**C**) Western blot of lysates from panel (**A**) was run on SDS PAGE. Protein was transferred to membrane a probed with anti-VP11 polyclonal sera (1:500 dilution). VP11 and VP16/VP17 expressed in *E. coli* were used as controls. The estimated size of proteins based on their sequences: VP11 = 22.12 KDa, VP16 = 19.11 KDa, and VP17 = ~32 KDa.

Analysis of bacterial lysates by SDS PAGE showed that VP11 (~22.1 Kda) and VP16 (~19.1 Kda, but not VP17) were expressed (Figure 2A). To confirm this, we performed Western blots on lysates using anti-VP11 polyclonal antibodies or a mixture of anti-VP16 and VP17 polyclonal antibodies (previously generated in our lab). As shown in Figure 2B,C, Western blot confirmed the expression of VP16 (and VP11 to some extent) but not VP17.

**Figure 3:**
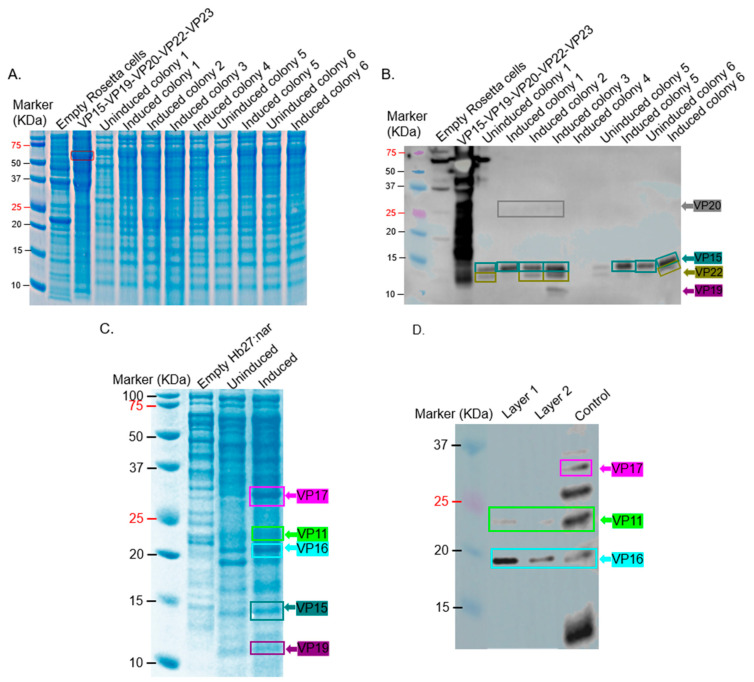
Co-expression of VP15/VP19/VP20/VP22/VP23 and co-expression of VP11/VP16/VP17 & VP15/VP19/VP20/VP22/VP23 in *T. thermophilus* HB27:nar. (**A**) Bacterial lysates (from co-expressed VP15/VP19/VP20/VP22/VP23 culture induced with 40 mM KNO_3_ for 4 h) were separated on SDS PAGE gel and stained with Coomassie Blue. (**B**) Lysates from panel (**A**) were run on SDS PAGE, and Western blot was conducted using anti-VP15/VP19/VP20/VP22/VP23 polyclonal sera (at 1:1000 dilution). (**C**) Bacterial lysates from co-expressed constructs (pMKE2-VP11/VP16/VP17 and pMKE2-VP15/VP19/VP20/VP22/VP23 induced with 40 mM KNO_3_ for 4 h) were separated on SDS PAGE gel and stained with Coomassie Blue. (**D**) Supernatants of samples from panel (**C**) were run on cesium chloride density gradients (1.14 g/mL and 1.27 g/mL). The samples were spun as described in the text, and layers were collected and analyzed for coat proteins. Layers were used for Western blot analysis using a mixture of anti-VP11, anti-VP16/VP17, and anti-VP15/VP19/VP20/VP22/VP23 recombinant protein polyclonal antibodies (at 1:500, 1:1000, and 1:1500, respectively). Control refers to the supernatant prior to ultracentrifugation. The estimated size of proteins based on their sequences: VP11 = 22.12 KDa, VP15 = 14.74 KDa, VP16 = 19.11 KDa, and VP17 = ~32 KDa, VP19 = 8.99 KDa, VP20 = 24.27 KDa, VP22 = 9.17 KDa, and VP23 = 7.26 KDa.

Regarding the co-expression of VP15/VP19/VP20/VP22/VP23, no expression was evident in SDS PAGE gel (Figure 3A). To confirm this, we performed a Western blot on lysates using polyclonal sera (Supplemental Figures S1–S3) from mice immunized with the recombinant protein (VP15-VP19-VP20-VP22-VP23) as primary antibodies. Lysates of VP15-VP19-VP20-VP22-VP23 recombinant protein (~65 KDa) expressed in *E. coli* were used as a positive control. Strong bands of ~14.7 KDa (expected size for VP15) were observed in induced samples, while less intense bands were observed in uninduced samples (Figure 3B). Also, in some of the induced samples, a less-intense band was observed below the 14.7 KDa band; we think this may be VP22 (which has a predicted size of ~9.2 KDa). In addition to these, a faint band at ~10 KDa was observed in the induced sample, which we believe corresponds to VP19. Furthermore, another very faint band was observed at ~25 KDa in the induced sample, which we believe corresponds to VP20 (with an expected size of ~24.3 KDa). To assess if coat proteins and membrane-associated proteins could be co-expressed, with the ultimate goal of assessing assembly, we co-transformed pMKE2-VP11/VP16/VP17 and pMKE2-VP15/VP19/VP20/VP22/VP23 into *T. thermophilus*. SDS PAGE analysis of bacterial lysates seemed to show the expression of VP11, VP15, VP16, VP17, and VP19 (Figure 3C). Analysis, via Western blot, of layer 1 from ultracentrifugation of the samples confirmed the expression of only VP11 and VP16 (Figure 3D).

### 2.2. VP11, VP15, VP16, VP17, VP19, VP20, and VP22 Were Successfully Co-Expressed in a Strain of E. coli (BL21 Star)

**Figure 4:**
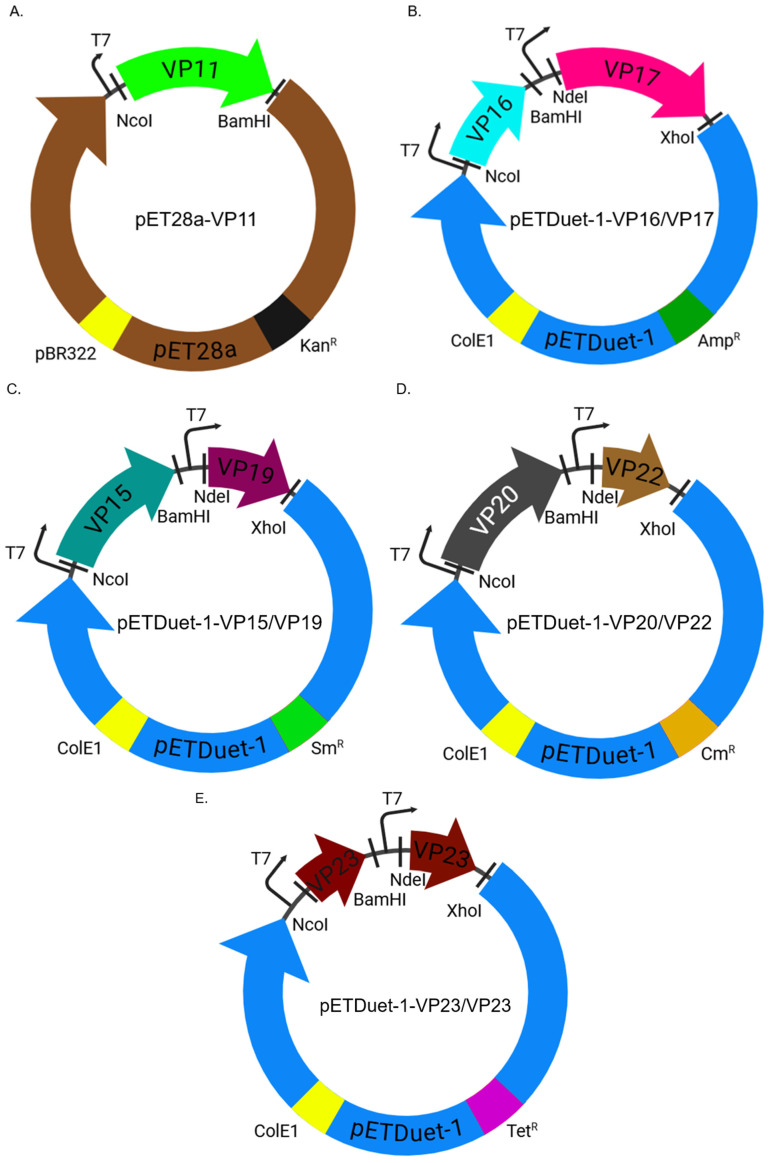
Design of the expression vectors of VPs in *E. coli* bacteria. (**A**) VP11 was cloned into pET28a (with the kanamycin resistance gene) using the indicated restriction sites. (**B**) VP16 and VP17 were cloned into pETDuet-1 (with the ampicillin resistance gene) using the indicated restriction sites and multiple cloning sites (MCS) I and II, respectively. (**C**) VP15 and VP19 were cloned into pETDuet-1 (a modified pETDuet-1 vector conferring streptomycin resistance) using the indicated restriction sites and MCS I and II, respectively. (**D**) VP20 and VP22 were cloned into pETDuet-1 (a modified pETDuet-1 vector conferring chloramphenicol resistance) using the indicated restriction sites and MCS I and II, respectively. (**E**) VP23 was cloned twice into pETDuet-1 (a modified pETDuet-1 vector conferring tetracycline resistance) in both MCS I and II using the restriction sites. T7: T7 promoter. pBR322 and ColE1: origins of replication. Kan^R^, Amp^R^, Sm^R^, Cm^R^, and Tet^R^: kanamycin, ampicillin, streptomycin, chloramphenicol, and tetracycline resistance genes, respectively.

Given the lack of expression of some of the membrane-associated proteins in *T. thermophilus* HB27:nar bacterium, we decided to explore expression in *E. coli* bacteria. To facilitate the co-expression of these proteins in a bacterium, the proteins were cloned in pairs in an expression vector. VP16 and VP17 were separately cloned together in pETDuet-1 (ampicillin resistant), VP15 and VP19 were separately cloned together in the same vector (a modified pETDuet-1 vector conferring streptomycin resistance), VP20 and VP22 were separately cloned together in the same vector (a modified pETDuet-1 vector conferring chloramphenicol resistance), VP23 was separately cloned in the same vector (a modified pETDuet-1 vector conferring tetracycline resistance), and VP11 was cloned into pET28a (kanamycin resistance) (Figure 4A–E).

**Figure 5:**
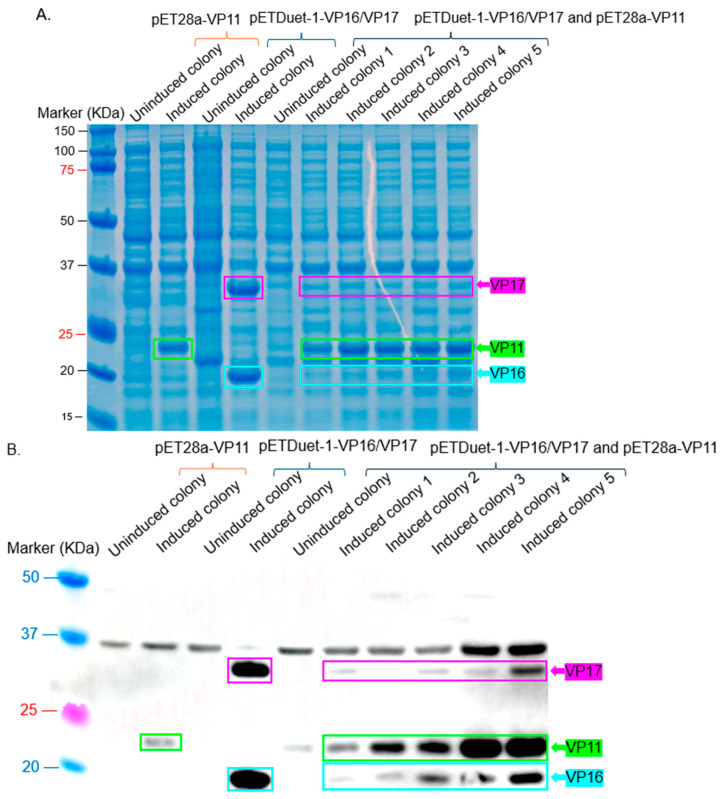

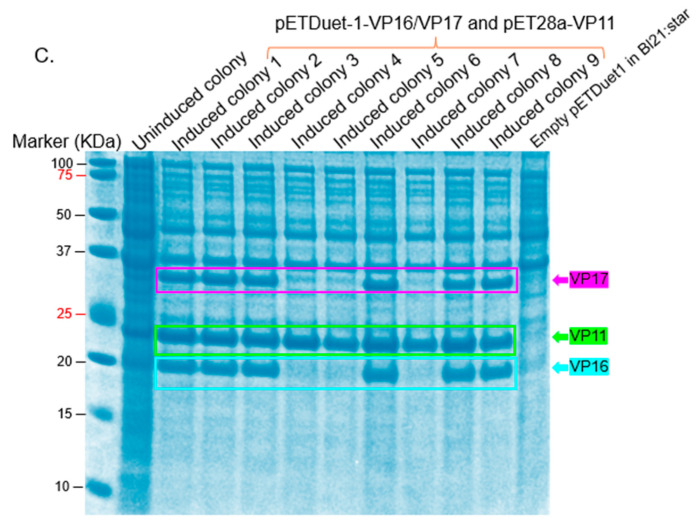
Co-expression of VP11 (pET28a) and VP16/VP17 (pETDuet-1) in *E. coli* Rosetta and BL21 Star. (**A**) SDS PAGE of lysates from co-expression of VP11 and VP16/VP17 in Rosetta 2 cells. (**B**) Western blot of lysates from panel (**A**). (**C**) SDS PAGE of lysates from co-expression of VP11 and VP16/17 in BL21 Star. The estimated size of proteins based on their sequences: VP11 = 22.12 KDa, VP16 = 19.11 KDa, and VP17 = ~32 KDa.

To check for expression of the coat proteins (VP11, VP16, and VP17), pET28-VP11 and pETDuet-1-VP16/VP17 were co-transformed into Rosetta 2 cells or BL21 Star cells (both are strains of *E. coli*) for the comparison of expression levels. All three coat proteins were co-expressed in the two strains of *E. coli* (Figure 5). However, the expression levels of VP16 and VP17 were very low in Rosetta 2 cells (Figure 5A); their expression could only be confirmed by Western blot (Figure 5B), unlike in BL21 Star, where expression was directly visible on the SDS PAGE gel (Figure 5C).

**Figure 6:**
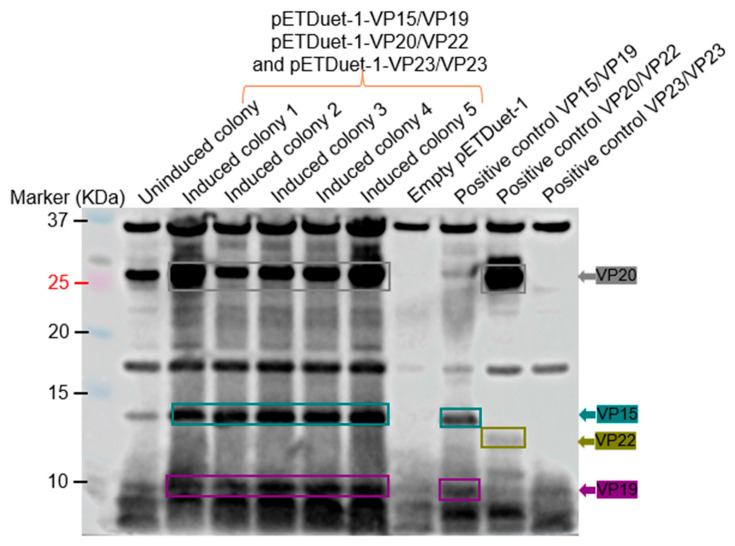
Western blot of co-expression of VP15/VP19, VP20/22 and VP23 in Bl21 star. Lysates of bacteria expressing the indicated proteins were run on SDS PAGE, and proteins were transferred to a membrane. Membranes were probed at 1:1500 dilution of VP15-VP19-VP20-VP22-VP23 recombinant protein polyclonal sera. Positive controls represent the expression of each construct individually under the same experimental conditions. Uninduced colony: bacteria containing three expression plasmids but without induction. An empty pETDuet-1 sample was included as a negative control, which represents the vector without target genes but induced. The estimated size of proteins based on their sequences: VP11 = 22.12 KDa, VP15 = 14.74 KDa, VP16 = 19.11 KDa, and VP17 = ~32 KDa, VP19 = 8.99 KDa, VP20 = 24.27 KDa, VP22 = 9.17 KDa, and VP23 = 7.26 KDa.

To assess co-expression of the membrane-associated proteins (VP15, VP19, VP20, VP22, and VP23), pETDuet-1-VP15/VP19, pETDuet-1-VP20/VP22, and pETDuet-1-VP23 were co-transformed into BL21 Star cells (given the fact that high expression levels were observed with the coat proteins). Only the expression of VP20 was evident in SDS PAGE analysis. Western blot confirmed this in addition to the expression of VP15 and VP19 (to an extent; Figure 6).

**Figure 7:**
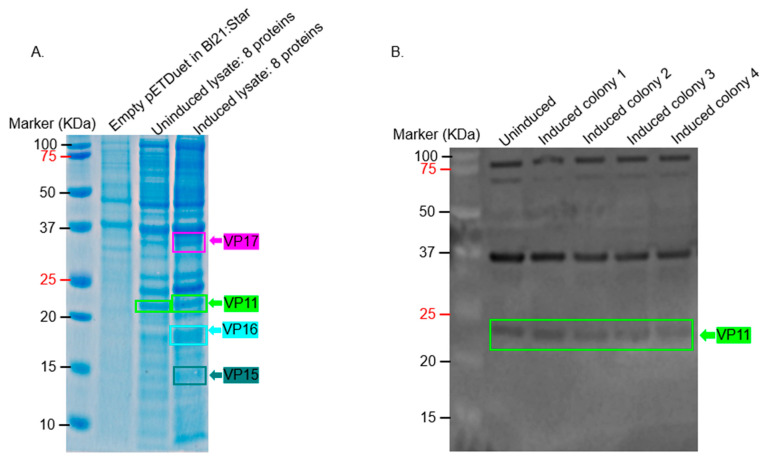

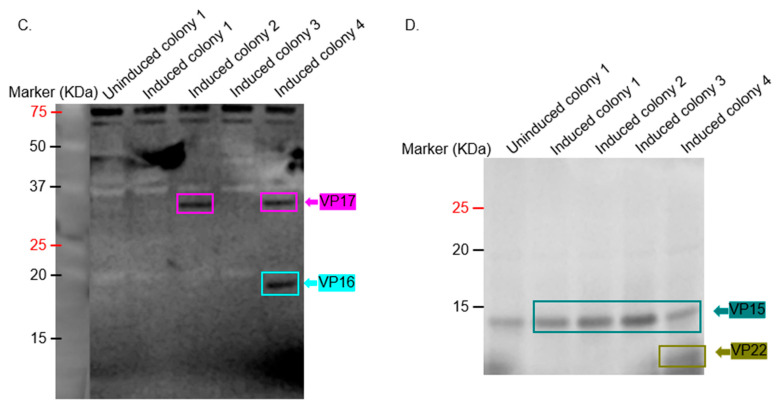
Co-expression of VP11, VP16/VP17, VP15/VP19, VP20/VP22, and VP23 in Bl21:star. (**A**) BL21 star cells were transformed with plasmids pET28-VP11, pETDuet-1-VP16/VP17, pETDuet-1-VP15/VP19, pETDuet-1-VP20/VP22, and pETDuet-1-VP23. Lysates of bacteria expressing proteins were run on SDS PAGE gel and stained with Coomassie Blue. Lysates from samples in panel (**A**) were run on SDS PAGE, and Western blot was conducted using 1:500 dilution of anti-VP11 polyclonal sera (**B**), 1:1000 dilution of a mixture of anti-VP16 and VP17 polyclonal sera (**C**), and 1:1500 dilution of anti-VP15-VP19-VP20-VP22-VP23 recombinant protein polyclonal sera (**D**). The estimated size of proteins based on their sequences: VP11 = 22.12 KDa, VP15 = 14.74 KDa, VP16 = 19.11 KDa, VP17 = ~32 KDa, VP19 = 8.99 KDa, VP20 = 24.27 KDa, VP22 = 9.17 KDa, and VP23 = 7.26 KDa.

To determine if the coat proteins and membrane-associated proteins (eight proteins altogether) could be co-expressed in the same cell with the ultimate goal of assessing assembly, we transformed BL21 Star with all five plasmids (Figure 4A–E) with five different antibiotic resistance genes. As shown in Figure 7A, VP11, VP15, VP16, and VP17 could be co-expressed. Western blot (Figure 7B–D) confirmed the expression of these proteins except VP15 and VP11, which also had bands in uninduced samples.

**Figure 8:**
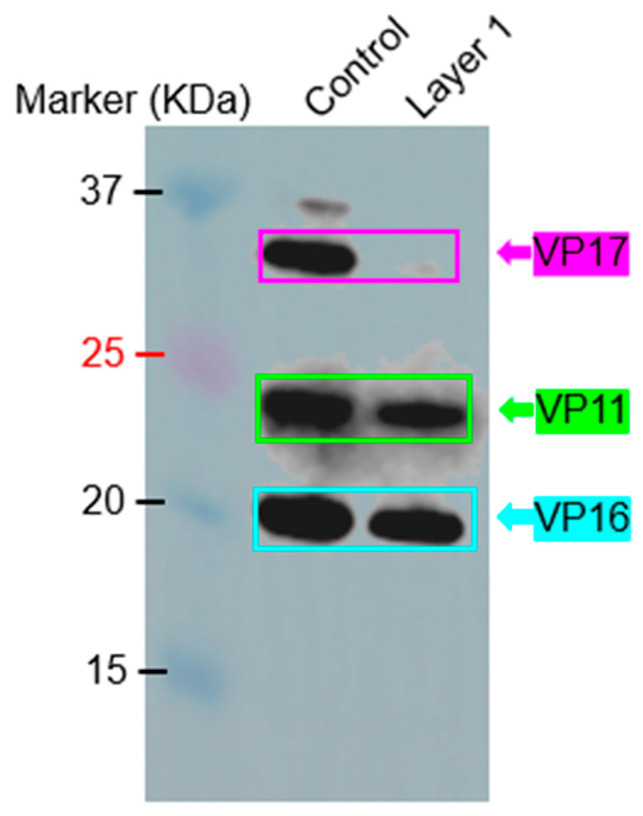
Cesium chloride gradient ultracentrifugation of proteins expressed in Bl21 Star. Bacterial cells expressing proteins were lysed with Bugbuster buffer, and the supernatant was loaded on 1.14 g/mL and 1.27 g/mL cesium chloride density gradients. The tubes were spun as described in the text, and the layers were analyzed for coat proteins via Western blot using a mixture of anti-VP11 and anti-VP16-VP17 polyclonal antibodies (at 1:500 and 1:1000, respectively). Control represents the supernatant before loading on the cesium chloride gradient. The estimated size of proteins based on their sequences: VP11 = 22.12 KDa, VP16 = 19.11 KDa, and VP17 = ~32 KDa.

To evaluate if these co-expressed proteins could assemble into VLPs, supernatants of co-expressed proteins were separated on a cesium chloride gradient column, and layers were analyzed using Western blot and transmission electron microscopy (TEM). Of all the three layers analyzed by Western blot, only layer 1 had two proteins, VP11 and VP16 (Figure 8). TEM analysis of this fraction showed oval structures (Supplemental Figure S4), but the average size (55 nm) was less than the expected size (78 nm).

### 2.3. VP11, VP15, VP16, VP17, VP19, and VP20 Were Successfully Purified from Bacterial Lysates, and Their Sizes Were Confirmed with Those Observed Above

**Figure 9:**
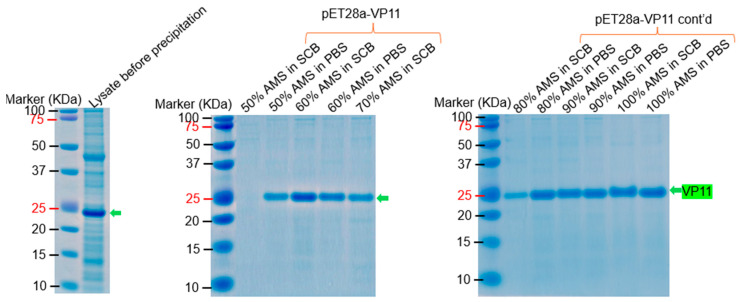
SDS PAGE of precipitation of VP11 from bacterial lysates with different percentages of ammonium sulfate (AMS). Bacterial pellet with VP11 expression was lysed with protein extraction reagent followed by two separate lysis with either SCB or PBS buffer (supplemented with 0.5% Triton X-100). AMS was added to the final supernatant at different concentrations (40–120%). The mixture was incubated in ice for 30 minutes, and the samples were spun at 10,000 rpm for 10 minutes. Pellets were resuspended in respective buffers, and samples were run on SDS PAGE gels. The estimated molecular weight of VP11 is approximately 22.12 KDa.

To evaluate whether some of these proteins can be purified and to further confirm the sizes observed above, we explored different strategies to purify these proteins. While some of the proteins were soluble in lysis buffer (and did not need a purification tag), some of the proteins required denaturing conditions to solubilize them and a purification tag for efficient purification. For example, VP11, VP16, and VP17 were relatively soluble after lysis in BugBuster protein extraction reagent, PBS, or SCB buffer, unlike the other proteins (VP15, VP19, and VP20 solubilized using urea). Attempts to purify VP11 via ion-exchange chromatography (based on its isoelectric point, 10.63) were unsuccessful. The addition of different percentages of ammonium sulfate to supernatant lysed with PBS buffer or SCB buffer (supplemented with 0.5% Triton-X 100) precipitated VP11 out of other contaminating proteins (Figure 9); the best precipitation was observed using 60–100% ammonium sulfate.

**Figure 10:**
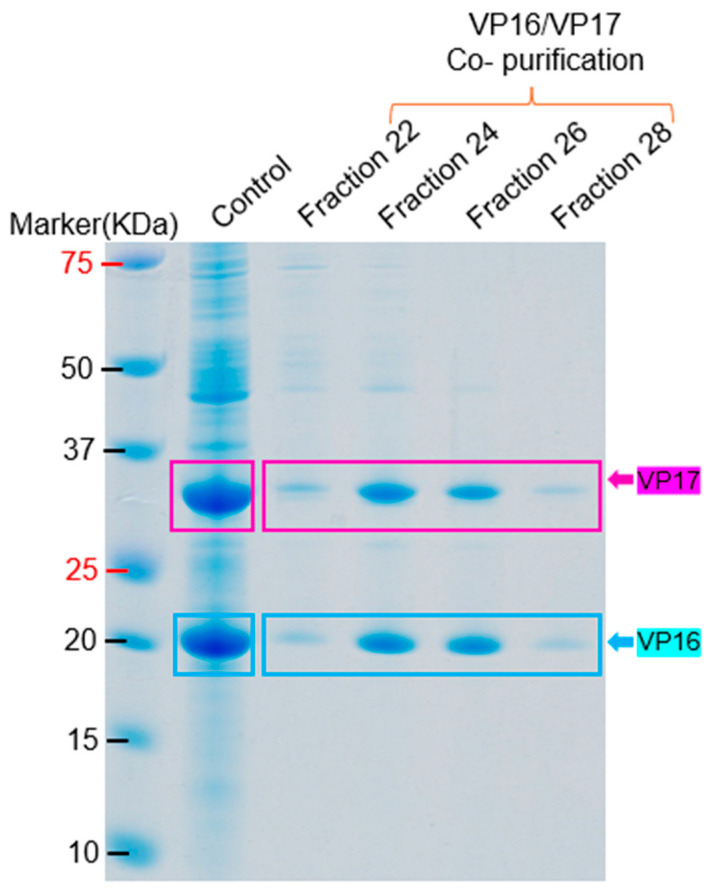
SDS PAGE of selected fractions of size exclusion chromatography of VP16/VP17. Supernatant of bacteria expressing VP16/VP17 was loaded on Sepharose CL-4B column; fractions were collected and assessed on SDS PAGE gel. Control represents the supernatant before loading on the column. The estimated molecular weight of VP16 and VP17 is approximately 19.11 and ~32 KDa, respectively.

For VP16 and VP17, the proteins were successfully co-purified via gel filtration on a Sepharose CL-4B column (Figure 10). The purified VP11, VP16, and VP17 migrated at ~22 KDa, ~19 KDa, and ~32 KDa. While the apparent co-elution of VP16 and VP17 may seem unexpected given their size difference, this could be due to several factors. First, protein migration in gel filtration chromatography (GFC) is influenced not only by size but also by shape; protein–protein interaction can affect any of these. It is possible that VP16 interacted non-covalently with VP17 during co-expression, leading to shared elution fractions. Furthermore, we performed ammonium sulfate (AMS) precipitation before GFC to enrich specific protein populations, which may have contributed to the co-purification of these two proteins [12,13].

**Figure 11:**
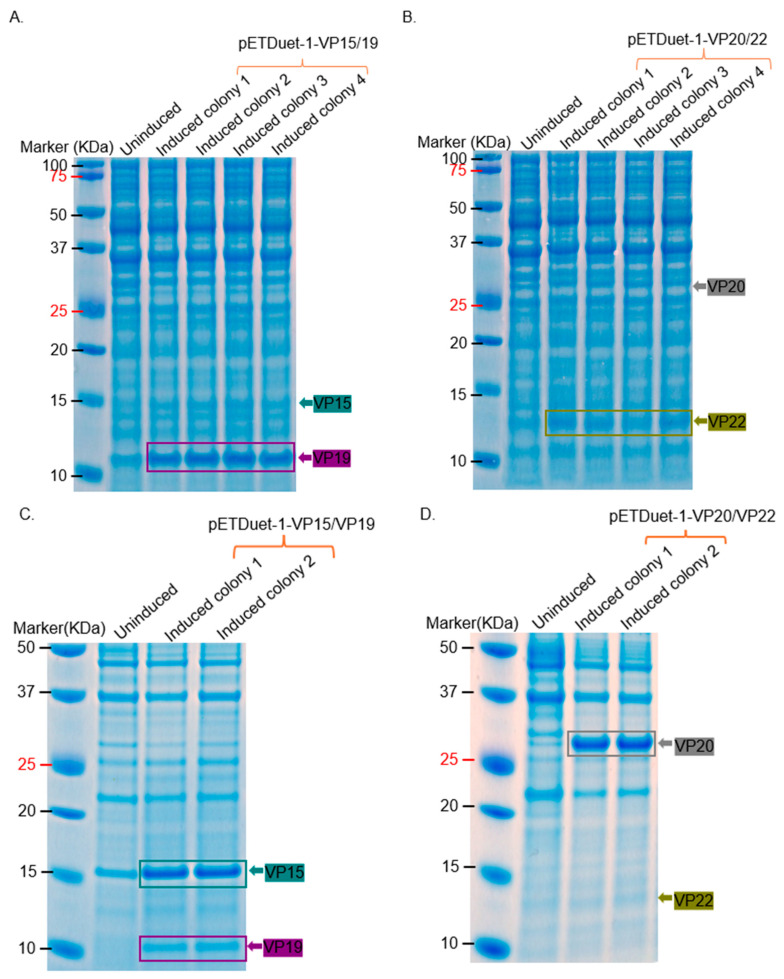
Expression of VP15, VP19, VP20, and VP22 proteins tagged with either His or Strep affinity tags in *E. coli* BL21 Star. (**A**) Co-expression of VP15-Strep-tag II and VP19-His-tag from pETDuet-1. (**B**) Co-expression of VP20-Strep-tag II and VP22-His-tag. (**C**) Co-expression of VP15-His-tag and VP19-Strep-tag II. (**D**) Co-expression of VP20-His-tag and VP22-Strep-tag II. The estimated sizes of proteins based on their sequences, including affinity tags and TEV cleavage site: VP15 = 16.72 KDa, VP19 = 11.04 KDa, VP20 = 25.3 KDa, and VP19 = ~11.0 KDa.

For the membrane-associated proteins, a purification tag was attached to the proteins for purification. The addition of Strep-tag II to the N-terminus of VP15 and VP20 reduced expression levels compared to the addition of his-tag to VP19 and VP22 (Figure 11A,B). Thus, new constructs were developed by swapping the tags. As shown in Figure 11C,D, the addition of his-tag to VP15 and VP20 increased their expression, while the addition of Strep-tag II to VP19 and VP22 decreased expression. Hence, attempts to purify these proteins were made using only those that had his-tag, even though they were co-expressed with those that had Strep-tag II.

**Figure 12:**
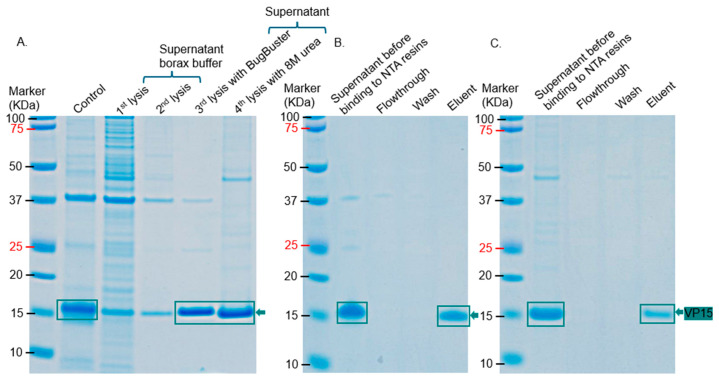
SDS PAGE of the purification of VP15-his-tag. (**A**) Pellet of bacteria co-expressing VP15-his-tag and VP19-Strep-tag II was lysed sequentially (twice in borax) and BugBuster reagent, followed by lysis with 8 M urea. Control represents the lysate of the same pellet, directly lysed with BugBuster reagent. (**B**) Purification of VP15-his-tag under native conditions [from the BugBuster supernatant (after 3rd lysis with BugBuster) in panel (**A**)] using Ni-NTA resin. Protein was eluted with a buffer that has 250 mM imidazole. (**C**) Purification of VP15 under denaturing conditions [from the 8 M urea supernatant (after 4th lysis with 8 M urea in panel (**A**)] using Ni-NTA resin. Protein was eluted with a buffer that has 1 M imidazole. The estimated molecular weight of VP15-his-tag, including the his-tag, is approximately 16.72 KDa.

While VP15-his-tag was soluble to an extent in Bugbuster lysis buffer, purified protein using this buffer had a contaminating band at ~37 KDa. However, when the bacterial pellet was lysed twice sequentially in borax buffer (before purification), this contaminating band was removed. As shown in Figure 12, the protein was successfully purified to homogeneity after resuspending the pellet, from two rounds of borax lysis, in either Bugbuster lysis buffer or 8 M urea. The purified protein migrated at ~16 KDa, which is close to the predicted size (16.72 KDa) of the protein with his-tag and TEV cleavage site inclusive (~2 KDa).

**Figure 13:**
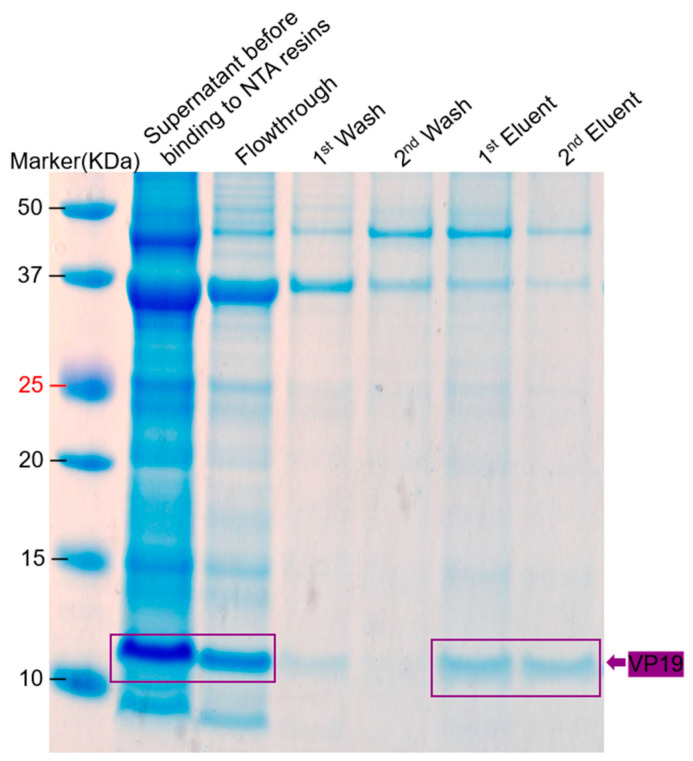
SDS Page gel of the purification of VP19-his-tag. Pellet of bacteria co-expressing VP19-his-tag and VP15-Strep-tag II was lysed in lysis buffer with 8 M urea, then bound to Ni-NTA resin. The resins were washed with 8 M urea lysis buffer supplemented with 5 mM imidazole (1st wash) and 10 mM imidazole (2nd wash). Protein was eluted using elution buffer (lysis buffer supplemented with 8 M urea and 250 mM imidazole; 1st eluent) and elution buffer (lysis buffer supplemented with 8 M urea and 500 mM imidazole; 2nd eluent). Elution was performed using the same buffer supplemented with 250 mM (1st elution) and 500 mM (2nd elution) imidazole. The estimated molecular weight of VP19, including his-tag, is approximately 11.0 KDa.

VP19-his-tag Protein was also successfully purified, although its purity was not good (Figure 13). With a his-tag and TEV cleavage site inclusive, the protein migrated at ~11–12 KDa, which is close to the size it migrated without a tag (at ~10 KDa; Figure 3B and Figure 6).

**Figure 14:**
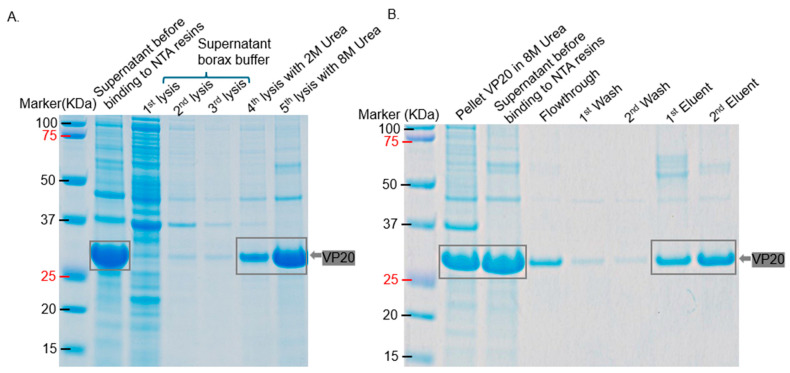
SDS Page gel of the purification of VP20-his-tag. Pellet of bacteria co-expressing VP20-his-tag and VP22-Strep-tag II was lysed with different lysis buffers. (**A**) VP20-expressing bacteria were sequentially lysed three times in borax buffer, followed by lysis in 2 M urea buffer and finally in lysis buffer containing 8 M urea. (**B**) The final supernatant was purified on Ni-NTA resin under denaturing conditions. VP20-his-tag was eluted using lysis buffer supplemented with 500 mM (1st elution) and 1 M (2nd elution) imidazole. The estimated molecular weight of VP20, including the his-tag, is approximately 25.3 kDa.

For VP20 purification, VP20-his-tag was not soluble in Bugbuster. Three sequential lysis of the pellet with borax buffer removed a lot of background protein. As shown in Figure 14, the protein was purified to homogeneity after resuspending the final pellets of borax buffer and 2 M urea in 8 M urea. The predicted size of VP-20-his-tag (with TEV cleavage site inclusive) is 25.3 KDa, but it migrated at >27 KDa.

### 2.4. Most of the Membrane-Associated Proteins Are Helical in Nature with Disordered Regions

**Figure 15:**
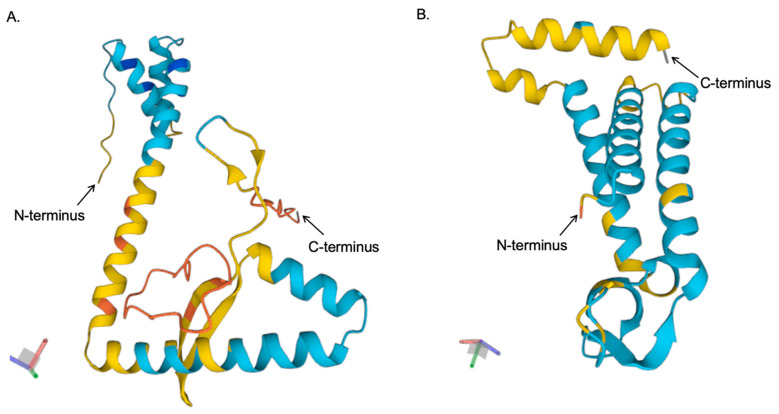
Predicted structures of VP11 and VP15 proteins. Amino acid sequences of VP11 and VP15 were input into ESM Metagenomic Atlas (https://esmatlas.com, accessed on 29 August 2025), and the results for (**A**) VP11 and (**B**) VP15 were generated with the “fold sequence” option. Local prediction confidence (pLDDT) score < 50 is shown in orange (considered very low confidence prediction), pLDDT score 50–69 is shown in yellow (considered low confidence prediction), pLDDT score > 70–80 is shown in light blue (considered a confident prediction), and pLDDT score > 90 is shown in dark blue (considered as very high confidence prediction).

To gain further insights into the potential structural characteristics of the membrane-associated proteins, we performed in silico 3D structure predictions (to assess the structures of the monomeric form of the proteins) using the ESM Metagenomic Atlas. VP11 and VP15 are made up of mostly helical and beta sheets with a few disordered regions (Figure 15), while VP19, VP20, VP22, and VP23 are made up of more helical and disordered regions (Supplemental Figure S5). The local prediction confidence (pLDDT) score per amino acid location for the proteins ranged from a low of 38 (very few amino acids) to a high of 91; the majority of residues in the structures had a pLDDT score > 50, except VP22 and VP23. A pLDDT score of <50 indicates a very low confidence prediction. It is worth mentioning that the predicted structures using the ESM Metagenomic Atlas were similar to those using AlphaFold3.

## 3. Discussion

As mentioned earlier, the three coat proteins (VP11, VP16, and VP17) of bacteriophage P23-77 have been previously expressed separately and purified from *E. coli* [5,14,15]. A mixture of these three proteins formed only oligomeric complexes and not VLPs [5]. The expression of other proteins (membrane-associated proteins: VP15, VP19, VP20, VP22, and VP23) known to associate with the coat proteins has never been explored, nor their potential to contribute to the formation of VLPs. Here, we assessed the expression of both coat proteins and membrane-associated proteins for the first time in the natural host bacterium, *T. thermophilus*, which the virus normally infects. The first goal was to assess the expression of the genes as they are organized in the genome of the virus (with their respective linker and native Shine–Dalgarno sequences) and also to assess their expression in an environment and conditions (temperature, osmolarity, pH, and metabolism) that mimic those under which the proteins are normally expressed. The second goal was to assess their expression in *E. coli* and the ability of the expressed proteins to assemble into VLPs (in comparison with expression in *T. thermophilus*). The third goal was to explore strategies for purifying these proteins from bacterial lysates for downstream assays (out of the scope of this proposal) and to predict their 3D structures. Using a polycistronic construct, we successfully co-expressed VP11 and VP16 for the first time in the same vector in *T. thermophilus* HB27:nar bacteria (Figure 2). While VP16 expression was evident in colony 2 by SDS-PAGE, its corresponding Western blot band was not evident; the protein may not have been efficiently transferred to the membrane, may not have been denatured properly, or may have been degraded.

For VP15, VP19, VP20, and VP22, the proteins were co-expressed using a similar approach and host cells above (Figure 3). Induced VP11 and VP15 samples on Western blot had bands at expected sizes (22.1 and 14.7 KDa, respectively); although bands of similar sizes were present in uninduced samples, these bands were less intense (Figure 2C and Figure 3B). We believe these less-intense bands may be a leaky expression of these proteins. VP22, with a predicted molecular weight of ~9.2 KDa, migrated at ~14 KDa. VP22 is ~51% identical (in amino acids) to open reading frame (ORF)19 of a related bacteriophage (ΦIN93); ORF19 has a predicted size of 10.3 KDa. In our previous study [16], we observed that ORF19 migrated at ~14 KDa, thus confirming that the observed band in this study may be VP22. In addition to VP22 migrating at a bigger size than predicted, VP20 also migrated at a bigger size. As mentioned above, the predicted size of VP20 is ~24.3 KDa; however, it migrated to around 26 KDa (Figure 3B). VP20 is ~69% identical (in amino acids) to ORF17 with a predicted size of 23.3 KDa to bacteriophage ΦIN93. We also observed in our previous studies that ORF17 migrated at a larger size (~25 KDa) [16]. Thus, we are confident that the faint band observed here at ~26 KDa is VP20.

Interestingly, we observed that VP15, which showed clear expression when expressed individually (Figure 3B), was barely detectable when co-expressed with other membrane-associated or capsid proteins (Figure 3C,D). This trend was consistent with additional observations in Figure 5, where the co-expression of VP16 and VP17 alone resulted in higher expression levels (Figure 5A), but their expression decreased upon the inclusion of VP11 (Figure 5B), which was strongly expressed. These findings suggest that introducing additional plasmids or expression cassettes may impose a metabolic burden or cause competitive effects at the transcriptional or translational level, affecting the balanced expression of individual proteins. This highlights a critical consideration for future optimization of multi-protein expression systems aimed at assembling complex viral structures.

Similar to our previous study with ΦIN93 [16], the expression levels of these proteins were better in *E. coli* bacterial strains compared to *T. thermophilus* HB27:nar. *E. coli*-codon optimized VP11, VP15, VP16, VP17, VP19, VP20, and VP22 were expressed at high levels in BL21 Star cells compared to in Rosetta 2 cells (Figure 5 and Figure 6). The sizes of the expressed proteins (especially VP20 and VP22) in *E. coli* were identical to those expressed in *T. thermophilus* HB27:nar, confirming that these two proteins migrate at sizes bigger than their predicted amino acid sequences. Studies have shown that high proline levels in protein can slow its migration on an SDS PAGE gel [17,18,19]. Among the five membrane-associated proteins expressed, VP20, VP22, and VP23 proteins are composed of more than 10% proline residues. VP20 has a proline ratio of 14%, VP22 has a proline ratio of 10%, while VP23 has a proline ratio of 11%; VP22 migrated at ~14 KDa as opposed to its predicted size of 9.17 KDa (Figure 11A). Unfortunately, we did not observe the expression of VP23.

In an attempt to co-express all eight proteins in either *T. thermophilus* HB27:nar or BL21 Star cells, only the expression of VP11 and VP16 could be confirmed in *T. thermophilus* HB27:nar, while in BL21 Star bacteria, only the expression of VP11, VP16, and VP17 could be confirmed (by Western blots; Figure 3D and Figure 7C). Overall, these proteins were co-expressed from different plasmids in BL21 Star (pET28-VP11-kanamycin resistant, pETDuet-1-VP16/VP17-ampicillin resistant, pETDuet-1-VP15/VP19-streptomycin resistant, and pETDuet-1-VP20/VP22-chloranphenicol resistant) in bacteria using five different antibiotics. We observed that culturing the bacteria using the recommended concentrations of antibiotics (50 µg/mL each of ampicillin, kanamycin, and streptomycin; 25–50 µg/mL of chloramphenicol; and 5–25 µg/mL of tetracycline) inhibited bacterial growth. To overcome this limitation, we reduced the concentration of the antibiotic combination to 6 µg/mL each of ampicillin, kanamycin, and streptomycin; 3 µg/mL of chloramphenicol; and 1 µg/mL of tetracycline. Reduced concentrations of antibiotic combinations allowed low but sufficient growth for downstream protein expression and analysis. To ensure that all plasmids were maintained under these lower antibiotic concentrations, a quality control step was performed: the same culture was divided and exposed to each antibiotic at its standard recommended concentration. Only cultures containing the corresponding plasmid(s) continued to grow under these conditions, confirming that plasmid retention was not compromised by the lowered antibiotic selection pressure during co-expression.

Six (VP11, VP15, VP16, VP17, VP20, and VP22) out of the eight structural proteins were successfully purified from *E. coli*. VP11, VP16, and VP17 migrated at ~22 KDa, ~19 KDa, and ~32 KDa, respectively; this thus confirms the fact that the three proteins observed at these sizes in bacterial lysates (Figure 2, Figure 3C,D, Figure 5A–C, Figure 7 and Figure 8) are VP11, VP16, and VP17 proteins. For the purification of the membrane-associated proteins, we observed that the addition of a Strep-tag II sequence at the N-terminus of proteins significantly reduced the expression levels of these proteins compared to the addition of his-tag (Figure 11). The observed reduction in protein expression upon addition of the Strep-tag II at the N-terminus may be attributed to several molecular factors. This could be due to the hydrophobicity of the tag. Strep-tag II is 37.5% hydrophobic compared to his-tag (0%). Studies have shown that hydrophobic proteins have low expression [20], and the insertion of this tag or its location may be the reason for low expression. We believe that any of the above may have been associated with reduced protein expression in this study. Thus, Strep-tag II is not a suitable purification tag for expressing and purifying these proteins. We also observed that the structural proteins may not be purified using the same approach. For example, VP11 could be purified simply by adding ammonium sulfate to precipitate the proteins (Figure 9), while VP15 and VP20 required sequential lysis in borax and/or 8M urea followed by affinity chromatography to purify the proteins (Figure 12 and Figure 14). VP15-his-tag migrated at ~16 KDa, which is close to the predicted size (16.72 KDa) of the protein with the his-tag and TEV cleavage site included (~2 KDa); this confirms that the band (around 15 KDa) we observed above in Figure 3B and Figure 6 is actually VP15.

VP20-his-tag migrated at a higher molecular weight, the same as what we observed for the protein without his-tag. As highlighted in the results, the predicted size of VP-20-his-tag (including TEV cleavage site) is 25.3 KDa, while its size without the tag is ~24.3 KDa. VP-20-his-tag migrated at >27 KDa, while VP20 without the tag migrated at ~26 KDa (Figure 6). This thus confirms that they are the same proteins, and that the protein observed in Figure 6 is actually VP20. Based on predicted 3D structures, VP11 and VP15 are made mostly of helical and beta sheets with a few disordered regions compared to the other membrane-associated proteins. The pLDDT score for the majority of amino acids in both VP11 and VP15 is >50 (Figure 15), which suggests that the predicted structures have confidence that ranges from low (yellow) to high (dark blue). These structures were compared with those predicted by AlphaFold, and they were similar, thus suggesting that the structures predicted using the ESM Metagenomic Atlas program have the same value. Moreover, when we predicted (using this program) the 3D structures of VP16 and VP17, which have already been determined and published [4], the structures were also similar, thus further validating the program.

In summary, most of the proteins were successfully expressed/co-expressed and purified, and their sizes were confirmed. It is not clear whether the oval structures (~55 nm as opposed to 78 nm) of co-expressed protein (in BL21 Star; Supplemental Figure S4) observed under the TEM are incomplete assembly of the proteins into VLPs or not given the fact that Western blot analysis of layers (used for TEM) from ultracentrifugation showed the absence of VP17 (Figure 8), a critical component of the viral capsid. Therefore, further studies are necessary to evaluate this. Future studies can focus on the following areas: For expression in thermophilic bacteria, express each of the coat proteins or membrane-associated proteins separately to check if they can be expressed individually, and then try to co-express them together, as was performed in *E. coli*. For thermophilic bacteria and *E. coli*, it is important to express and purify the proteins separately, then mix them together under varying concentrations and different buffer conditions (e.g., ionic strength, pH, and presence of crowding agents or nucleic acids) to determine if they can assemble. It may also be important to evaluate whether membrane mimetics (such as liposomes or detergents) are necessary for proper folding or scaffolding of the membrane-associated proteins. Overall, the structures predicted here, while computational, are a good starting point for structural biologists to build upon and determine the 3D structures of the predictions.

## 4. Materials and Methods

### 4.1. Cloning of Coat Proteins and Membrane-Associated Proteins in Expression Vectors

DNA sequences corresponding to the open reading frames of coat proteins (VP11, VP16, and VP17), including an 11-base pair sequence in between VP11 and VP16 (5′ GGAGGTAAAGG 3′) and a 10-base pair sequence in between VP16 and VP17 (5′ GGAGGTGAGC 3′) that separate the two coat proteins, were synthesized by Epoch Life Sciences (Figure 1A). The nucleotides included part of a native Shine–Dalgarno sequence present in the genome of P23-77, which is upstream of VP16 and VP17. The DNA fragment was amplified by PCR, digested, and cloned into the pMKE2 vector (a gift from Dr. Jose Berenguer, Universidad Autónoma de Madrid); cloning was performed downstream of a respiratory nitrate reductase promoter (Pnar) using NcoI and EcoRI sites. The pMKE2 vector is a thermophilic vector that enables the expression of foreign proteins (from Pnar) in thermophilic bacteria [21,22].

To co-express the membrane-associated proteins (VP15, VP19, VP20, VP22, and VP23), a polycistronic construct that has DNA sequences of the five open reading frames, separated by 8–13-base pair sequence (with native Shine-Dalgarno sequences as they appear in the genome of P23-77), was also synthesized and cloned separately into the pMKE2 vector (Figure 1B), as described above. All plasmid constructs were sequenced across cloning junctions to confirm the authenticity of the genes.

To express the above proteins in *E. coli*, the genes that code for them were codon-optimized for *E. coli* expression. The codon-optimized genes were synthesized and cloned into bacterial expression vectors (by Epoch Life Sciences) for co-expression as follows: VP11 was cloned separately into the pET28a vector (kanamycin resistance) (Figure 4A). VP16 and VP17 were cloned, respectively, into multiple cloning site (MCS) 1 and 2 in pETDuet-1 vector (ampicillin resistance); VP15 and VP19 were cloned, respectively, into MCS1 and 2 in pETDuet-1 vector (a modified pETDuet-1 vector conferring streptomycin resistance); VP20 and VP22 were cloned, respectively, into MCS1 and 2 in pETDuet-1 vector (a modified pETDuet-1 vector conferring chloramphenicol resistance); and one copy of VP23 was cloned into MCS1 and another into MCS2 in pETDuet-1 vector (a modified pETDuet-1 vector conferring tetracycline resistance) (Figure 4B–E).

### 4.2. Co-Expression of Coat Proteins and Membrane-Associated Proteins in a Thermophilic Bacterium (HB27:nar) and in E. coli

To check if the coat proteins and membrane-associated proteins could be expressed, the polycistronic vectors (pMKE2-VP11/VP16/VP17 or pMKE2-VP15/VP19/VP20/VP22/VP23) were used to separately transform a thermophilic bacterium, *Thermus thermophilus* HB27:nar (also a gift from Dr. Jose Berenguer). HB27:nar is a derivative of the HB27 strain that carries a respiratory nitrate reductase (nar) operon, which allows the bacteria to also grow anaerobically [23]. Transformation was performed as follows: HB27:nar was grown overnight at 70 °C in Terrific broth medium (TB medium: 8 g of peptone, 4 g of yeast extract, 3 g of NaCl, pH 7.5). Overnight cultures were diluted in 1:50 fresh TB medium and grown at 200 rpm at 70 °C until an optical density (OD_600_) of 0.4 was reached. Eight hundred microliters of the cells were transformed separately with 300 ng of each of the plasmids and were cultured at 250 rpm at 70 °C for an additional 4 h. The transformants were concentrated to 100 µL (by centrifugation at 3000 rpm) and then plated on 3% Terrific broth agar plates containing 30 µg/mL of kanamycin; the plates were incubated at 70 °C for 2 to 3 days. To screen for protein expression, colonies were picked from the agar plates and individually inoculated into 5 mL of TB medium containing 30 µg of kanamycin. The mixture was grown at 70 °C (shaking at 250 rpm) until an OD_600_ of 0.4 was reached. Protein expression was induced by adding 40 mM potassium nitrate (KNO_3_), and the cells were anaerobically incubated (without shaking) at the same temperature for 4 h. To check for protein expression, cultures were pelleted and lysed, and the lysate/supernatant was run on SDS PAGE followed by Western blotting. Cultures that showed protein expression (of expected sizes) were used to isolate plasmids for co-expression of the two plasmids (pMKE2-VP11/VP16/VP17 and pMKE2-VP15/VP19/VP20/VP22/VP23). Co-expression of the proteins from two plasmids was performed by mixing the two plasmids in equal concentrations (each construct was 150 ng) and transforming HB27:nar bacteria as described above using the same antibiotic concentration. Protein expression and induction were performed as described above.

To co-express the proteins in *E. coli*, pET28a-VP11 vector (expressing VP11), pETDuet-1-VP16/VP17 vector (expressing VP16 and VP17), pETDuet-1-VP15/VP19 vector (expressing VP15 and VP19), pETDuet-1-VP20/VP22 vector (expressing VP20 and VP22), and pETDuet-1-VP23/VP23 vector (expressing VP23) were used to separately transform Rosetta 2(DE3)pLysS or BL21 Star(DE3) to screen and compare protein expression levels. Following the confirmation of protein expression by SDS-PAGE and/or Western blots, all five expression vectors (each carrying a different antibiotic resistance marker) were used to co-transform BL21 Star(DE3) cells. Transformed cells were cultured in Luria Bertani media with a five-antibiotic combination: 6 µg/mL each of ampicillin, kanamycin, and streptomycin; 3 µg/mL of chloramphenicol; and 1 µg/mL of tetracycline.

### 4.3. Ultracentrifugation of Co-Expressed Proteins to Assess the Formation of VLPs

For expression in *thermophilic bacteria* (HB27:nar) and *E. coli* (BL21 Star), lysates of bacteria from co-expression of all eight proteins (VP11, VP16, VP17, VP15, VP19, VP20, VP22, and VP23) were spun at 10,000 rpm for 10 minutes. Their supernatants were run on cesium chloride density gradients (1.14 g/mL and 1.27 g/mL). The samples were centrifuged at 20,000 rpm for 16 h (4 °C), and different layers were collected for SDS-PAGE, Western blot, and TEM analysis.

### 4.4. Western Blot on Bacterial Lysates and on Layers from Ultracentrifugation

Lysates from HB27:nar bacteria transformed/co-transformed with pMKE2-VP11/VP16/VP17 and pMKE2-VP15/VP19/VP20/VP22/VP23. Lysates from BL21 Star bacteria were transformed/co-transformed with pET28a-VP11 vector, pETDuet-1-VP16/VP17 vector, pETDuet-1-VP15/VP19 vector, pETDuet-1-VP20/VP22 vector, and pETDuet-1-VP23/VP23 vector; resolved on SDS-PAGE gels; and transferred onto polyvinylidene difluoride membranes. Layers from ultracentrifugation of supernatants derived from the lysates were also resolved and transferred to the membranes. The PVDF membranes were blocked using blocking buffer with 5% milk/TBST, and 1:500 to 1:1000 dilutions of VP11 mixture, 1:500 to 1:1000 dilutions of VP16 and VP17 mixture, and VP15-VP19-VP20-VP22-VP23 recombinant protein polyclonal sera 1:1500 (generated in our lab; Supplementary Figures S1–S3) were added and incubated for 2 h. Horseradish peroxidase-conjugated goat anti-mouse IgG antibodies (1:10,000 dilution) were added to the membranes and incubated for 1 h. The membranes were washed and developed using SuperSignal West Pico (Thermo Fisher, Waltham, MA, USA) (Luminol/Enhancer and stable peroxide) solutions.

### 4.5. Transmission Electron Microscopy (TEM)

To evaluate whether the coat proteins could self-assemble into virus-like particles (VLPs), transmission electron microscopy (TEM) was performed on samples collected from ultracentrifugation layers, as shown by Western blot to contain viral proteins. These samples were applied to glow-discharged carbon-coated grids and incubated for 2 minutes. The grids were then negatively stained with 2% uranyl acetate for 2 minutes. Imaging was carried out using a Hitachi H-7650 (Hitachi, Tokyo, Japan) transmission electron microscope.

### 4.6. Purification of Coat Proteins and Membrane-Associated Proteins from E. coli Lysates to Confirm Sizes

Each of the coat proteins was expressed and purified separately from *E. coli* as follows. For the expression and purification of VP11, VP16, and VP17, the constructs shown in Figure 4A,B were used. For the expression and purification of VP15 and VP19, Strep-tag II (WSHPQFEK, a purification tag) was inserted into the N-terminus of VP15, while eight histidine residues (his-tag, another purification tag) were inserted to the N-terminus of VP19 in the construct shown in Figure 4C; in between each purification tag, a TEV cleavage site (ENLYFQS) was included, for future use, to enable cleavage of the tag after purification. Other constructs were developed whereby the his-tag was inserted on the N-terminus of VP15, while Strep-tag II was inserted into the N-terminus of VP19. A similar approach was used to express and purify VP20 and VP22. Strep-tag II and his-tag were each inserted into the N-terminus of VP20 and VP19 in the construct shown in Figure 4D. For VP23, his-tag was inserted into each of the VP23 constructs in Figure 4E.

For purification, the bacterial pellet expressing VP11 was lysed with Bugbuster protein extraction reagent, centrifuged, and the pellet was lysed twice in either Sepharose column buffer (SCB) or phosphate-buffered saline (PBS) buffer (supplemented with 0.5% Triton-X-100). Different concentrations (40–120%) of ammonium sulfate were added to the supernatant to precipitate VP11. For VP16 and VP17 purification, the bacterial pellet expressing the proteins was lysed with SCB buffer, and the supernatant was run on a Sepharose CL-4B column; protein fractions were collected and analyzed on an SDS PAGE gel.

For the purification of VP15, the bacterial pellet expressing the protein was lysed twice (sequentially) in borax buffer (100 mM Sodium tetraborate decahydrate). The resulting pellet was then lysed using Bugbuster reagent, followed by an additional round of lysis in 8 M urea. Each lysate (from Bugbuster and 8 M urea) was centrifuged, and its supernatant was used for purification using Ni-NTA resin as follows. The supernatant was incubated with Ni-NTA resin for 1 h. The resins were pelleted by centrifugation at 2000 rpm for 2 minutes, washed three times with lysis buffer (20 mM sodium phosphate, 50 mM NaCl, 0.5% Triton X-100, 1 mM DTT, 5% glycerol, 20 mM imidazole) for native purification, or washed three times with lysis buffer (supplemented with 8 M urea) for denatured purification. VP15 was eluted from the resins by adding elution buffer (lysis buffer with 250 mM imidazole) for native purification or by adding elution buffer (lysis buffer with 8 M urea and 1 M imidazole) for denatured purification; the resins were incubated with the buffer for 1 h and centrifuged at 2000 rpm for 2 minutes, and eluted protein in the buffer was collected.

For VP19 purification, the bacterial pellet expressing the protein was lysed in lysis buffer containing 8 M urea and then bound to Ni-NTA resin. The resins were washed with 8 M urea lysis buffer supplemented with 5 mM imidazole (1st wash) and 10 mM imidazole (2nd wash). Protein was eluted using elution buffer (lysis buffer supplemented with 8 M urea and 250 mM imidazole; 1st eluent) and elution buffer (lysis buffer supplemented with 8 M urea and 500 mM imidazole; 2nd eluent).

For the purification of VP20, the bacterial pellet expressing the protein was lysed three times (sequentially) in borax buffer, followed by lysis in 2 M urea buffer and finally in lysis buffer with 8 M urea. Protein was added to Ni-NTA resin, incubated, and washed, as described above. VP20 was eluted using elution buffer (lysis buffer with 8 M urea supplemented with 500 mM imidazole; 1st eluent) and elution buffer (lysis buffer with 8 M urea supplemented with 1 M imidazole; 2nd eluent).

### 4.7. Prediction of the 3D Structures of Membrane-Associated Proteins

The three (3)-dimensional structures of membrane-associated proteins (VP11, VP15, VP19, VP20, VP22, and VP23) were predicted using the ESM Metagenomic Atlas (https://esmatlas.com, accessed on 29 August 2025). The original amino acid sequences of each protein were input into the ESM Atlas interface, which predicts tertiary structures from primary sequences by applying transformer-based protein language models.

## Data Availability

Additional data contained within this article are included in the Supplementary Materials.

## Supplementary Materials

The following supporting information can be downloaded at: https://www.mdpi.com/article/10.3390/ijms26178688/s1.

## References

[1] Yu M.X., Slater M.R., Ackermann H.W. (2006). Isolation and characterization of Thermus bacteriophages. Arch. Virol..

[2] Jaatinen S.T., Happonen L.J., Laurinmaki P., Butcher S.J., Bamford D.H. (2008). Biochemical and structural characterisation of membrane-containing icosahedral dsDNA bacteriophages infecting thermophilic Thermus thermophilus. Virology.

[3] Jalasvuori M., Jaatinen S.T., Laurinavicius S., Ahola-Iivarinen E., Kalkkinen N., Bamford D.H., Bamford J.K. (2009). The closest relatives of icosahedral viruses of thermophilic bacteria are among viruses and plasmids of the halophilic archaea. J. Virol..

[4] Rissanen I., Grimes J.M., Pawlowski A., Mantynen S., Harlos K., Bamford J.K., Stuart D.I. (2013). Bacteriophage P23-77 capsid protein structures reveal the archetype of an ancient branch from a major virus lineage. Structure.

[5] Pawlowski A., Moilanen A.M., Rissanen I.A., Määttä J.A., Hytönen V.P., Ihalainen J.A., Bamford J.K. (2015). The minor capsid protein VP11 of thermophilic bacteriophage P23-77 facilitates virus assembly by using lipid-protein interactions. J. Virol..

[6] Liu H., Kheirvari M., Tumban E. (2023). Potential Applications of Thermophilic Bacteriophages in One Health. Int. J. Mol. Sci..

[7] Yadav R. (2021). Efficacy of Mixed MS2-L2 VLPs Against Six HPV Types and the Development & Evaluation of Viral Structural Proteins for Assembly into VLPs. Ph.D. Thesis.

[8] Chroboczek J., Szurgot I., Szolajska E. (2014). Virus-like particles as vaccine. Acta Biochim. Pol..

[9] Zeltins A. (2013). Construction and characterization of virus-like particles: A review. Mol. Biotechnol..

[10] Schwarz B., Uchida M., Douglas T. (2017). Biomedical and Catalytic Opportunities of Virus-Like Particles in Nanotechnology. Adv. Virus Res..

[11] Demina T.A., Pietila M.K., Svirskaite J., Ravantti J.J., Atanasova N.S., Bamford D.H., Oksanen H.M. (2017). HCIV-1 and Other Tailless Icosahedral Internal Membrane-Containing Viruses of the Family Sphaerolipoviridae. Viruses.

[12] Baker S.L., Munasinghe A., Kaupbayeva B., Rebecca Kang N., Certiat M., Murata H., Matyjaszewski K., Lin P., Colina C.M., Russell A.J. (2019). Transforming protein-polymer conjugate purification by tuning protein solubility. Nat. Commun..

[13] Marceau A.H. (2012). Ammonium sulfate co-precipitation of SSB and interacting proteins. Methods Mol. Biol..

[14] Rissanen I. Purification and Crystallization of the Two Major Coat Proteins of Bacteriophage P23-77 for X-Ray Crystallography 2009. https://jyx.jyu.fi/bitstream/handle/123456789/23246/1/URN%3ANBN%3Afi%3Ajyu-201004191538.pdf.

[15] Rissanen I., Pawlowski A., Harlos K., Grimes J.M., Stuart D.I., Bamford J.K. (2012). Crystallization and preliminary crystallographic analysis of the major capsid proteins VP16 and VP17 of bacteriophage P23-77. Struct. Biol. Cryst. Commun..

[16] Liu H., Kheirvari M., Tumban E. (2025). Design, Co-Expression, and Evaluation for Assembly of the Structural Proteins from Thermophilic Bacteriophage PhiIN93. Int. J. Mol. Sci..

[17] Liu Y., Zhou J. (2024). The P124A mutation of SRP14 alters its migration on SDS-PAGE without impacting its function. Acta Biochim. Biophys. Sin..

[18] Kirkland T.N., Finley F., Orsborn K.I., Galgiani J.N. (1998). Evaluation of the proline-rich antigen of Coccidioides immitis as a vaccine candidate in mice. Infect. Immun..

[19] Stainier I., Bleves S., Josenhans C., Karmani L., Kerbourch C., Lambermont I., Totemeyer S., Boyd A., Cornelis G.R. (2000). YscP, a Yersinia protein required for Yop secretion that is surface exposed, and released in low Ca^2+^. Mol. Microbiol..

[20] van Gils J.H.M., Gogishvili D., van Eck J., Bouwmeester R., van Dijk E., Abeln S. (2022). How sticky are our proteins? Quantifying hydrophobicity of the human proteome. Bioinform. Adv..

[21] Moreno R., Haro A., Castellanos A., Berenguer J. (2005). High-level overproduction of His-tagged Tth DNA polymerase in Thermus thermophilus. Appl. Environ. Microbiol..

[22] Wu W.L., Liao J.H., Lin G.H., Lin M.H., Chang Y.C., Liang S.Y., Yang F.L., Khoo K.H., Wu S.H. (2013). Phosphoproteomic analysis reveals the effects of PilF phosphorylation on type IV pilus and biofilm formation in Thermus thermophilus HB27. Mol. Cell. Proteom..

[23] Ramirez-Arcos S., Fernandez-Herrero L.A., Marin I., Berenguer J. (1998). Anaerobic growth, a property horizontally transferred by an Hfr-like mechanism among extreme thermophiles. J. Bacteriol..

