# Supplementary material for: The Structural Proteins of Thermophilic Bacteriophage P23-77: Expression and Characterization
Source: Int J Mol Sci. 2025 Sep 6;26(17):8688. doi: 10.3390/ijms26178688 (PMC12429598; doi:10.3390/ijms26178688)
Supplement: Supplementary file 1 [file ijms-26-08688-s001.zip › ijms-3832184-supplementary.pdf]

Supplementary Information

Supplemental Materials and Methods

Expression and purification of VP15-VP19-VP20-VP22-VP23 recombinant protein

VP15, VP19, VP20, VP22, and VP23 genes were codon-optimized for bacterial expression. The genes were synthesized as one open reading frame fragment without start and stop codons between each of the ORFs; three glycine residues were included between each of the ORFs to enhance flexibility of individual proteins (supplemental Fig S1). Six-histidine residues were added to the C-terminus of the gene fragment for affinity purification. The recombinant protein was cloned into pET28a vector using NcoI/BamHI sites. The recombinant protein was transformed into Rosetta cells; the protein was expressed and purified as previously described [1].

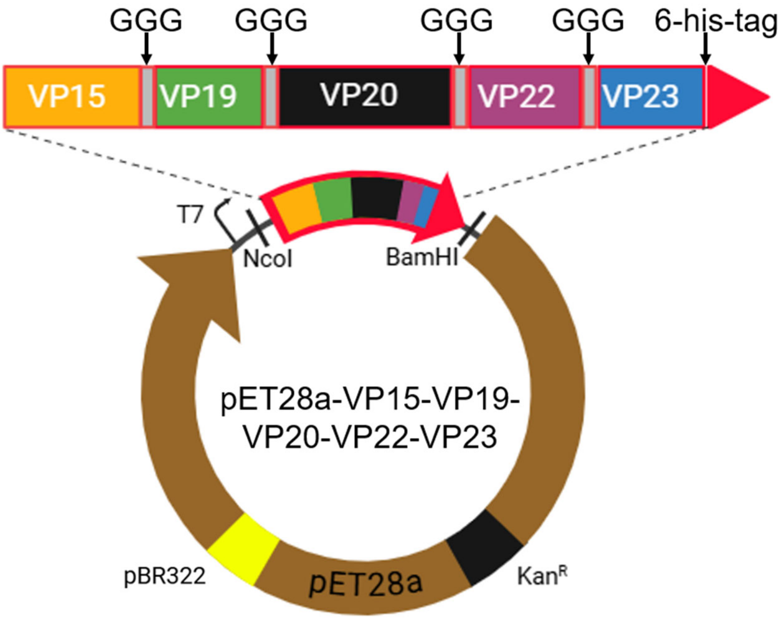

Supplemental Fig. S1. Design of the expression of VP15-VP19-VP20-VP22-VP23 recombinant protein in pET28a. The gene fragment was cloned using NcoI/BamHi restriction sites. T7: T7 promoter. pBR322: origins of replication. Kan^R is Kanamycin resistance gene.

Generation of polyclonal antibodies against VP15-VP19-VP20-VP22-VP23 recombinant protein

Animal work was approved by Texas Tech University Health Sciences Center IACUC (Institutional Animal Care and Use Committee). To generate polyclonal antibodies, 10μg of VP15-VP19-VP20-VP22-VP23 recombinant protein was used to immunize Balb/c mice subcutaneously; all immunizations were done with alum adjuvant, and mice were immunized 3 times at two-week intervals. Two weeks after the last immunization, whole blood was collected from mice and antibody titers (total IgG) in sera were determined using the recombinant protein as target antigens.

Supplementary Results

VP15-VP19-VP20-VP22-VP23 recombinant protein was successfully purified from bacterial lysates. The protein migrated at the expected size of ~65 KDa (Supplemental Fig. S2). Mice immunized with the purified VP15-VP19-VP20-VP22-VP23 recombinant protein elicited very high titer IgG antibody response (geometric mean >10^5) compared to control naïve sera (Supplemental Figs. S3). TEM analysis of layer 1 from density gradient ultracentrifugation showed oval structures with an average size of ~55 nm (Supplemental Fig. S4)

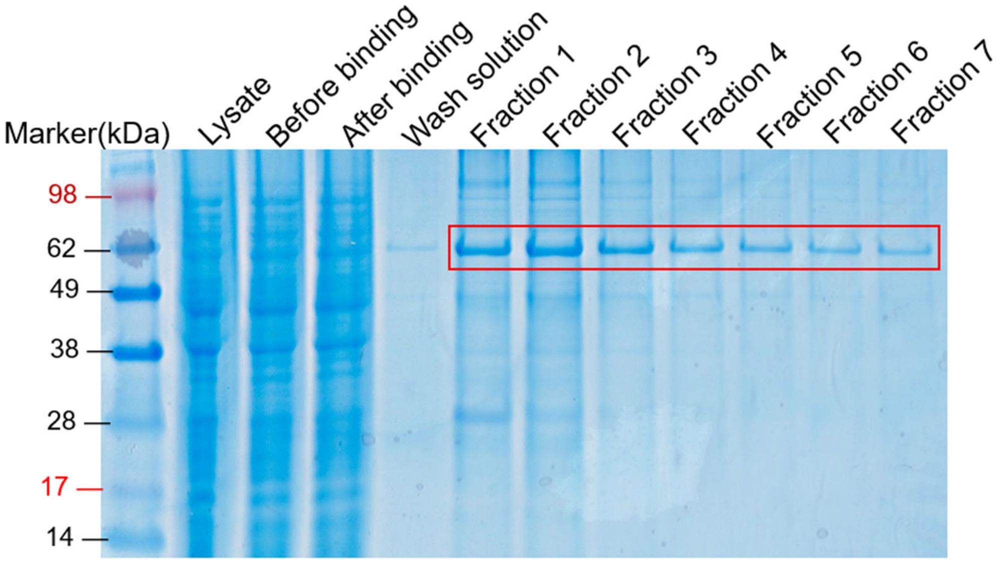

Supplemental Fig. S2: Expression and purification of VP15-VP19-VP20-VP22-VP23 recombinant protein in Rosetta cells. Rosetta cells transformed with the vector expressing the recombinant protein were induced with 0.5 mM IPTG. Cells were grown for 4 additional hours, lysed with 2M urea and the protein was purified on a nickel NTA column. Fractions were collected and run on a 10% SDS PAGE gel followed by staining with Coomassie blue dye.

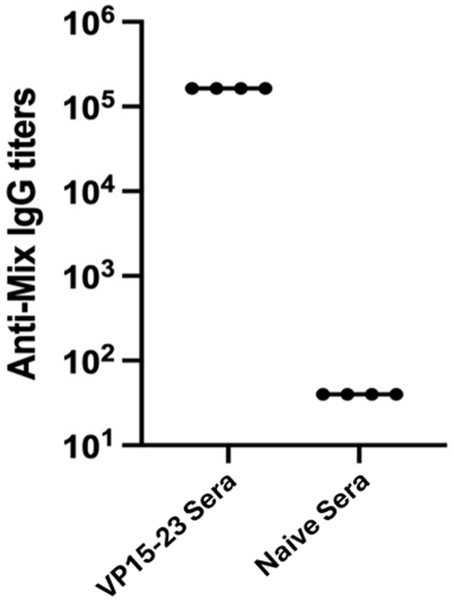

Supplemental Fig. S3: Immunogenicity of VP15-VP19-VP20-VP22-VP23 recombinant protein. Balb/c mice were immunized thrice by the recombinant protein including VP15, VP19, VP20, VP22, and VP23. Whole blood was collected from mice and IgG titers were determined by ELISAs using the protein as target antigen.

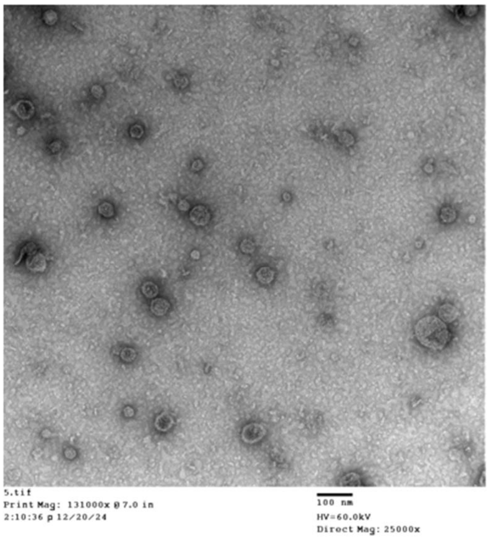

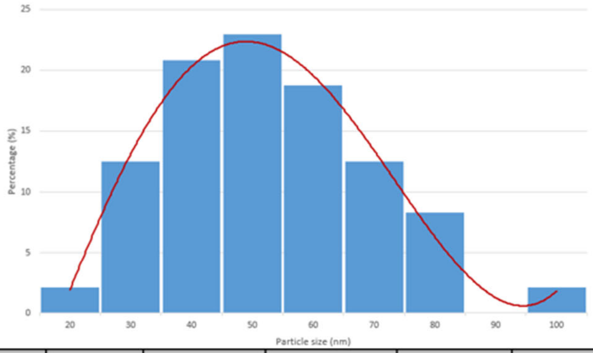

Mean	Median	Minimum	Maximum	Std Dev	Std Error
52.71	50	21.5	105	16.85	2.43

Supplemental Fig. S4: TEM analyses. Samples from layer 1 of ultracentrifugation of supernatants from proteins expressed in BL21 Star were loaded onto glow-discharged carbon grids for 2 minutes, stained with 2% uranyl acetate and visualized using a transmission electron microscope. Graph on the right is a size distribution of the oval structures from TEM.

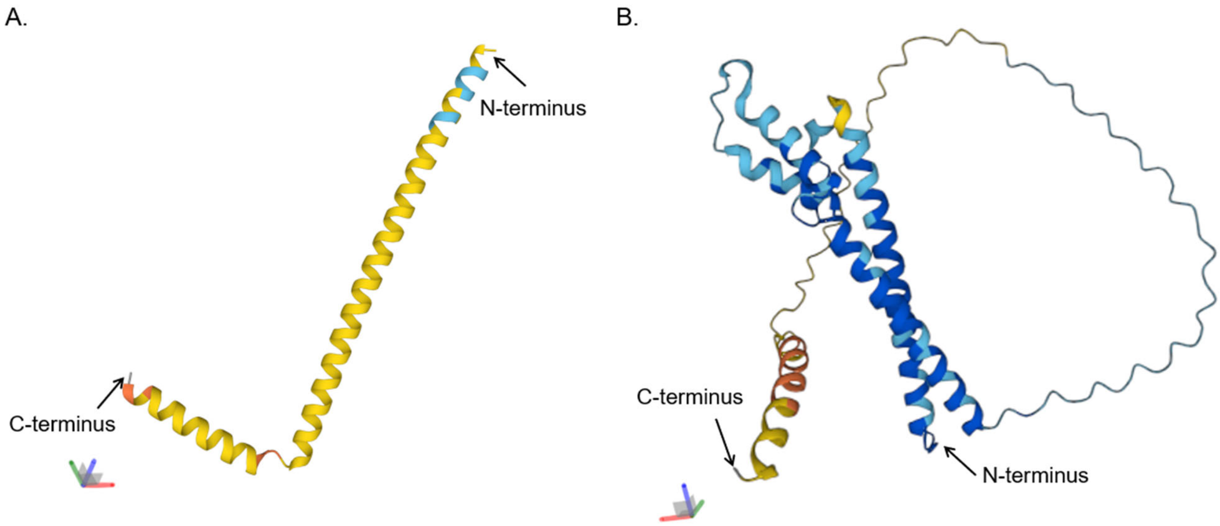

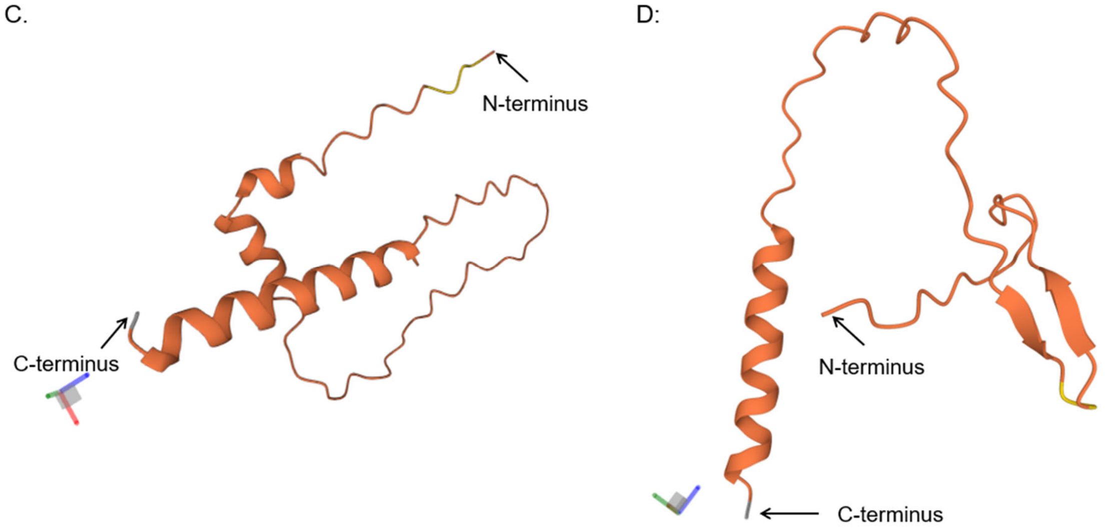

Supplementary Fig. S5: Predicted structures of VP19, VP20, VP22, and VP23 proteins. Amino acid sequences of these proteins were put into ESM Metagenomic Atlas (https://esmatlas.com) and the results for VP19 (A), VP20 (B), VP22 (C), and VP23 (D) were generate with the “fold sequence” option. Local prediction confidence (pLDDT) score <50 is shown in orange (considered very low confidence prediction), pLDDT score 50-69 is shown in yellow (considered low confidence prediction), pLDDT score >70-80 is shown in light blue (considered a confident prediction), pLDDT score >90 is shown in dark blue (considered as very high confidence prediction).

Reference:

1. Liu, H., M. Kheirvari, and E. Tumban, *Design, Co-Expression, and Evaluation for Assembly of the Structural Proteins from Thermophilic Bacteriophage PhiIN93.* Int J Mol Sci, 2025. **26**(11).
